# Circular RNA expression profile in peripheral blood mononuclear cells from Crohn disease patients

**DOI:** 10.1097/MD.0000000000016072

**Published:** 2019-06-28

**Authors:** Juan Yin, Tong Hu, Lijuan Xu, Ping Li, Meifen Li, Yulan Ye, Zhi Pang

**Affiliations:** a Digestive Disease and Nutrition Research Center; b Department of Gastroenterology, The Affiliated Suzhou Hospital of Nanjing Medical University, Suzhou, Jiangsu, China.

**Keywords:** circular RNAs, Crohn disease, inflammatory bowel disease, microarray analysis, peripheral blood mononuclear cells

## Abstract

Crohn disease (CD) is a multifactorial autoimmune disease which is characterized by chronic and recurrent gastrointestinal tract inflammatory disorder. However, the molecular mechanisms of CD remain unclear. Increasing evidences have demonstrated that circular RNAs (circRNAs) participate in the pathogenesis of a variety of disease and were considered as ideal biomarkers in human disease. This study aimed to investigate circRNA expression profiles and detect new biomarkers in inflammatory bowel disease (IBD). Differentially expression of circRNAs between CD and HCs (health controls) were screened by microarray analysis. Peripheral blood mononuclear cells (PBMCs) from 5 CD patients and 5 HCs were included in the microarray analysis. Then, the differences were validated by quantitative polymerase chain reaction (qPCR) following reverse transcription polymerase chain reaction (RT-PCR) in the patients of CD and sex- and age-matched HCs. The most differential expressed circRNA was further validated in ulcerative colitis (UC) patients. Statistical significance between CD, UC, and HCs was analyzed by Student *t* test for unpaired samples or one-way analysis of variance (ANOVA). Diagnostic value of each circRNA was assessed by receiver operating characteristic (ROC) curve. We identified 155 up-regulated circRNAs and 229 down-regulated ones by microarray analysis in PBMCs from CD patients compared with HCs. Besides, 4 circRNAs (092520, 102610, 004662, and 103124) were significantly up-regulated validated by RT-PCR and qPCR between CD and HCs. ROC curve analysis suggested important values of circRNAs (092520, 102610, 004662, and 103124) in CD diagnosis, with area under the curve (AUC) as 0.66, 0.78, 0.85, and 0.74, respectively. Then, we further identified that the relative expression levels of circRNA_004662 was upregulated significantly in CD patients compared with UC patients. Herein, the upregulation of the 4 circRNAs (092520, 102610, 004662, or 103124) in PBMCs can be served as potential diagnostic biomarkers of CD, and circRNA_004662 might be a novel candidate for differentiating CD from UC. Moreover, a circRNA–microRNA-mRNA network predicted that circRNA_004662 appeared to be correlated with mammalian target of rapamycin (mTOR) pathway.

## 1 Introduction

With rapid growth of incidence of Crohn disease (CD) and ulcerative colitis (UC) in newly industrialized countries in Asia, inflammatory bowel disease (IBD) has become popular worldwide.^[1,2]^ The etiology of IBD is complex, involving host genetic background, and environmental and microbial factors.^[3,4]^

CD is a chronic discontinuous disease of the gastrointestinal tract, and usually involves the colon or terminal ileum.^[5]^ On contrary, UC affects only the rectum and colon.^[6]^ To diagnose CD or UC specifically, clinical symptoms, endoscopic features, radiological tests, and biopsy histology should be all taken into consideration,^[6]^ and lack of early diagnosis can present with serious disease in IBD patients. However, there are no adequate sensitive diagnostic biomarkers for CD and UC. Thus, the identification of effective biomarkers for IBD is crucial.

However, blood serum markers, such as anti-neutrophil cytoplasm antibody (ANCA), anti-laminaribioside carbohydrate antibody (ALCA), and anti-Saccharomyces cerevisiae antibody (ASCA) with low specificity or sensitivity have limited values for initially diagnosis of IBD.^[7]^

Circular RNAs (circRNAs) are generated from back-spliced events of precursor mRNAs (pre-mRNAs) and circularization of exons, introns, or both exons, and introns.^[8]^ They are predominantly non-coding RNAs (ncRNAs) acting as regulatory elements. Increasing evidences demonstrated that circRNAs can modulate gene expression at the transcriptional or post-transcriptional level by sponging microRNAs (miRNAs) or by interrelating with other molecules.^[9]^ Furthermore, circRNAs are evolutionally conserved and expressed stable relatively in the cytoplasm,^[10]^ these features show that circRNAs may be ideal biomarkers in human disease.

Studies have shown that circRNAs play roles in the process of multiple diseases, such as colorectal cancer (CRC), breast cancer, rheumatoid arthritis (RA), and pre-eclampsia.^[11–17]^ In the field of digestive diseases, a number of studies associated with circRNAs have recently focused on CRC. Both down- and up-regulated circRNAs related to the process of CRC were detected by microarray screening.^[11,12]^ In CRC, the down-regulated circ_0014717 promotes expression of p16 to inhibit tumor growth.^[18]^ The progression of CRC is suppressed by circRNA_0026344 via microRNA-31 and microRNA-21 as well.^[19]^ Besides, downregulated hsa_circ_0001649 can be served as a novel biomarker of CRC for disease inspection.^[20]^ It is well-known that there are certain links between chronic inflammation and cancer.^[21]^ At present, it should be elucidated whether circRNAs play key roles in diagnosis of IBD, as few reports have focused on this topic yet.

The aim of this research was to discover novel potential diagnostic markers of IBD by identification of differentially expressed circRNAs in PBMCs of patients with CD and UC. Differences on circRNAs’ expression were preliminarily screened by microarray, and then validated by quantitative polymerase chain reaction (qPCR) following reverse transcription polymerase chain reaction (RT-PCR). Bioinformatic analysis was also used to predict possible sponging miRNA targets. A circRNA-miRNA-mRNA network was built by cytoscape.

## 2 Materials and methods

### 2.1 Sample collection

In this study, 5 patients of CD and 5 healthy controls were used for circRNA microarray analysis. Fifty-two CD patients and 38 HCs were applied for initial verification. The most differential expressed circRNA was further validated in 50 samples from UC patients and expanded samples (87 CD patients and 55 HCs). The age and sex of all patients were matched. The clinical features of the participants are shown in Table 1. The IBD patients and HCs were collected from the Suzhou Affiliated Hospital of Nanjing Medical University (Suzhou, Jiangsu province, China) from 2017 to 2018. All CD patients were recently diagnosed without any treatment, and assessed according to Crohn Disease Activity Index (CDAI).^[22]^ Patients who previously received antibiotic drug and therapeutic antibodies were excluded. Besides, an ethical agreement by the Ethics Committee of Nanjing Medical University was obtained prior to the start of this study. And informed consent forms from all the participants were received.

**Table 1:**
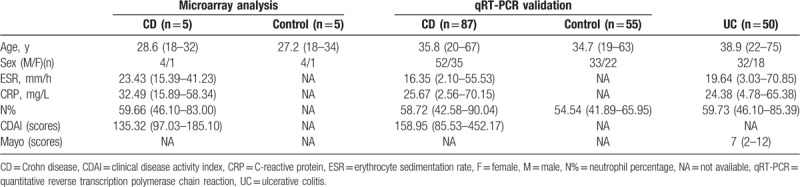
Clinical information of the participants.

### 2.2 Extraction of total RNA from PBMCs

Here, PBMCs were immediately separated performing after blood sample collection from each donor according to a protocol mentioned in the following. Two milliliter blood diluted in 2 mL saline solution was layered on 4 mL Ficoll-Paque PLUS (GE Healthcare, Uppsala, Sweden). After centrifugation for 30 minutes at 400 × *g* and at room temperature, the interlayer was collected by being washed with the same volume of saline solution twice. The precipitate was gathered by centrifuging for 15 minutes at 90 × *g* and at room temperature. PBMCs were then frozen at –80 °C. After that, TRIzol regent (Invitrogen, Carlsbad CA) was utilized for total RNA extraction from PBMCs.

### 2.3 Microarray hybridization and data analysis

The microarray hybridization of all the RNA samples was undertaken as previously described by Ouyang et al.^[15]^ Human circRNA Arrays (Arraystar Inc., Rockville, MD) was used for screening differentially expressed circRNAs.

Briefly, first, total RNAs were digested with RNase R to enrich circular RNAs. Second, the enriched circular RNAs were amplified and transcribed into fluorescent cRNAs with a random priming method according to the Arraystar Super RNA Labeling protocol (Arraystar, Inc). Then, the labeled cRNAs were hybridized on the Arraystar Human circRNA Array V2.0 (Arraystar, Inc), and incubated for 17 hours at 65 °C. Finally, the hybridized slides were washing and scanned with the Agilent Scanner G2505C (agilent United States California, Santa Clara, Agilent santa clara, CA). Raw data were extracted into Agilent Feature Extraction software (agilent United States California, Santa Clara, Agilent santa clara, CA). Quantile normalization and data processing were performed by the R software (https://www.r-project.org/). CircRNAs that were differentially expressed between the CD and HC groups were conveniently identified by fold-change cut-off value and Student *t* tests. The “fold-changes” of circRNA >2.0 and *P* < .05 were considered as significantly abnormal expressed.

Additionally, filtering the Volcano Plot, filtering the Fold-Change (FC), and the hierarchical clustering were carried out to identify circRNAs expression profiles of CD patients to distinguish from HCs. TargetScan and miRanda were used to predict the interaction between circRNA and miRNA.

### 2.4 Verification of significantly upregulated circRNAs

In this study, 5 circRNAs exhibiting up-regulated expression screened by microarray were selected for validation by RT-PCR and qPCR. Primers used have been shown in Table 2. Divergent primer design refers to Panda and Gorospe.^[23]^ Besides, PrimeScript Master Mix (TaKaRa, Shiga, Japan) was used to synthesize cDNA from total RNA. Then, circRNAs’ relative expression level was detected by TB Green Premix Ex Taq II (Tli RNaseH Plus; TaKaRa, Shiga, Japan) with β-actin as an internal control. The validation of all the circRNAs by qPCR were performed in a particular LightCycler 480 II (Roche, Rotkreuz, Switzerland). 2^–ΔΔ^ Ct method was used to analyze the data.^[24]^

**Table 2:**
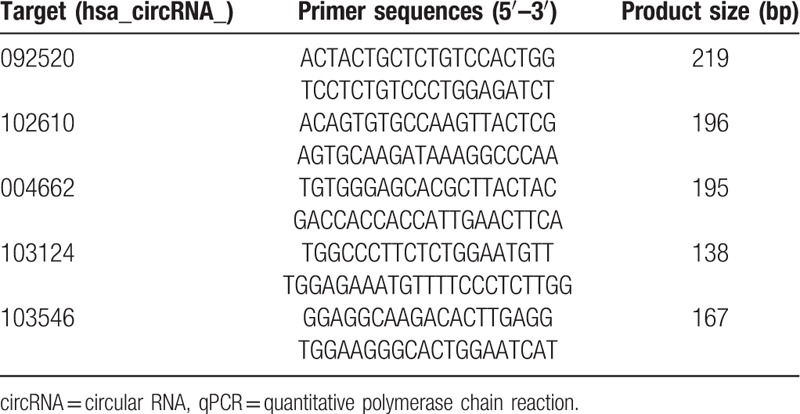
Primers used in qPCR for validation.

### 2.5 Statistical analysis

Statistical significance between groups was calculated by Student *t* test or one-way analysis of variance (ANOVA). The diagnostic value of circRNAs was assessed by receiver operating characteristic (ROC) curves. GraphPad Prism 7.0 (GraphPad Software, San Diego, CA) and SPSS statistics version19.0 software (SPSS, Inc, Chicago, IL) were used to analyze all the statistical data. *P* value <.05 was considered to be statistically significant.

## 3 Results

### 3.1 CircRNAs expression profiles of CD patients

**Figure 1:**
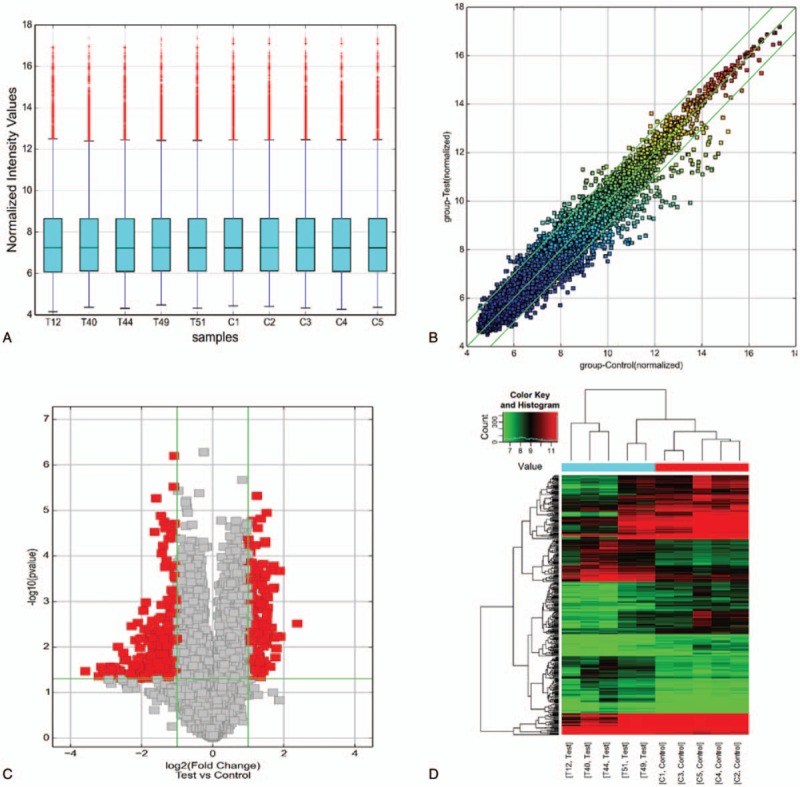
CircRNA expression profiles of CD patients (n = 5) in comparison with HCs (n = 5) screened by microarray analysis. A, Boxplot view showed the distribution of normalized intensity values for CD patients (T12, T40, T49, and T51) and HCs (C1, C2, C3, C4, and C5). B, Scatter plot indicated the variation of circRNA expression between groups. Dots outside the green lines represented circRNAs with logarithmized expression changes greater than 2-fold between groups. C, Volcano plots showed the values of FC and P of the microarray data. Red dots indicated significantly dysregulated circRNAs. D, Hierarchical clustering revealed circRNA expression profiles of CD and HCs. Red color strip represented relatively high expression, while green color one indicated relatively low expression. circRNA = circular RNA, CD = Crohn disease, FC = fold-change, HCs = health controls.

In our study, 155 up-regulated and 229 down-regulated circRNAs were identified in CD patients compared with HCs by microarray analysis. Information of circRNAs being classified as top 10 up-regulated or down-regulated ones have been listed in Table 3. Box plot view (Fig. 1A) showed distribution of expression values between CD patients and HCs. Filtering the FC (Fig. 1B) showed circRNAs expressed differentially between groups; filtering the Volcano Plot (Fig. 1C) showed circRNAs differentially expressed between groups with statistical significance; hierarchical clustering (Fig. 1D) showed distinguished circRNAs expression patterns among samples.

**Table 3:**
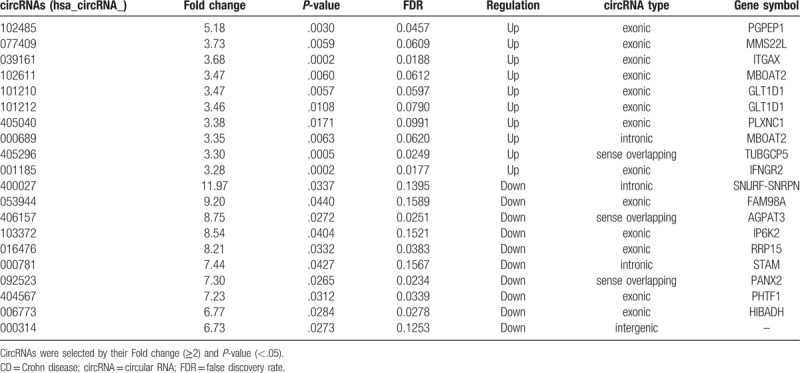
Top 10 up-regulated or down-regulated circRNAs in CD patients screened by microarray analysis.

### 3.2 Validating the results of differentially expressed circRNAs

**Figure 2:**
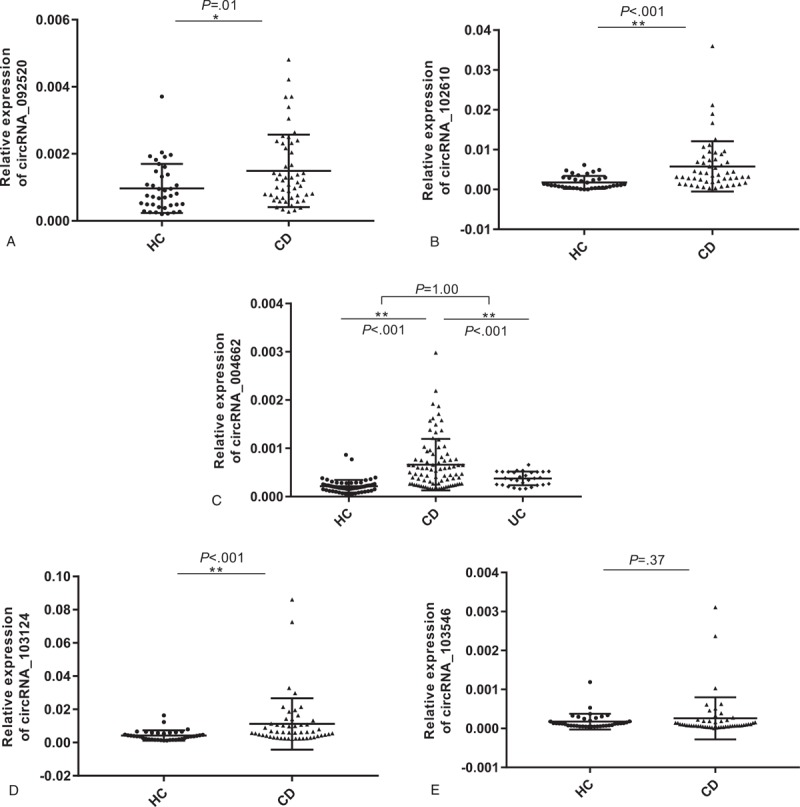
Verification of the relatively expression level of 5 up-regulated circRNAs (092520, 102610, 004662, 103124, and 103546) by RT-PCR and qPCR. PBMCs from 52 CD patients and 38 HCs were used for initial verification. CircRNA_004662 was further validated in 87 CD patients and 55 HCs and 50 UC patients. The statistical significance between CD, UC, and HCs was calculated by Student *t* test or one-way ANOVA. A, The relative expression of circRNA_09250. B, The relative expression of circRNA_102610. C, The relative expression of circRNA_004662. D, The relative expression of circRNA_103124. E, The relative expression of circRNA_103546. *P* < .05 was considered as statistically significant. ∗*P* = .01; ∗∗*P* < .001. ANOVA = analysis of variance, CD = Crohn disease,circRNA = circular RNA, HCs = health controls, qPCR = quantitative polymerase chain reaction, RT-PCR = reverse transcription polymerase chain reaction, PBMCs = peripheral blood mononuclear cells, UC = ulcerative colitis.

Here, 5 up-regulated circRNAs were chosen (Table 4) for further RT-PCR and qPCR detection, according to their Fold change (>2), *P*-values (<.05), and raw intensities (>200). PBMCs from 52 CD patients and 38 HCs were used for initial verification (see Fig. 2). The most differential expressed circRNA was further validated in 87 CD patients and 55 HCs and 50 UC patients. In line with the data of microarray, the relative expression levels of 4 circRNAs (092520, 102610, 004662, and 103124) were up-regulated significantly in CD patients compared with HCs. (As showed in Fig. 2A–D). Moreover, the relative expression level of circRNA_103546 showed that there was no remarkable difference between CD patients and HCs (Fig. 2E).

**Table 4:**
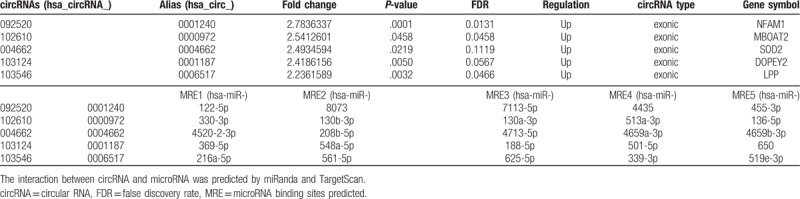
Microarray analysis of the 5 up-regulated circRNAs (validated).

Nevertheless, we found that the circRNA_004662 expression level in CD was up-regulated compared with UC (*P* < .001), but there was no difference between UC and HCs (*P* = 1.00) (Fig. 2C). Hence in, circRNA_004662 might be a novel candidate for differentiating CD from UC.

### 3.3 ROC analysis of validated circRNAs in CD patients

**Figure 3:**
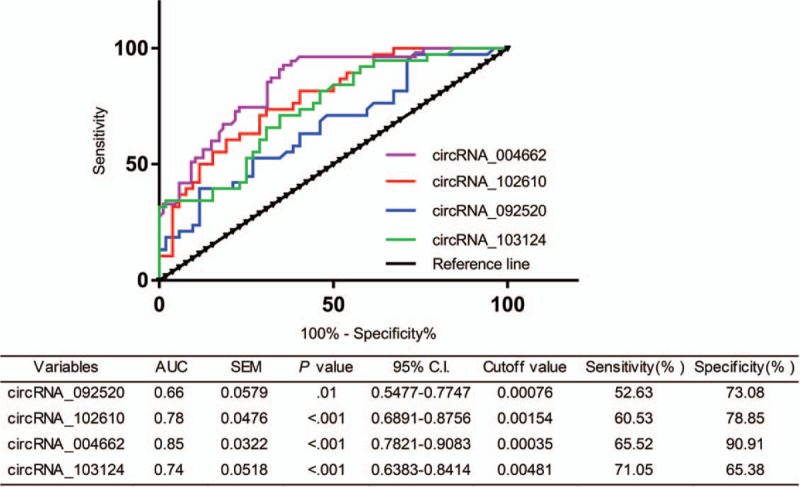
ROC curve analysis to evaluate diagnostic value of validated circRNAs. Four up-regulated circRNAs (103124, 004662, 102610, and 092520) verified were analyzed. AUC = area under curve, circRNA = circular RNA, CI = confidence interval, ROC = receiver operating characteristic, SEM = standard error of mean.

Possible diagnostic value of these significantly up-regulated circRNAs in CD patients was evaluated by ROC curve analysis. The achieved results are illustrated in Fig. 3. According to relative expression levels of any one of these 4 circRNAs (092520, 102610, 004662, or 103124) in PBMCs, the population of CD patients and HCs can be distinguished. The values of area under the ROC curve (AUC) of these 4 circRNAs were 0.66, 0.78, 0.85, and 0.74 respectively. Considering values of AUC, *P*-value, sensitivity, and specificity (see Fig. 3), circRNA_004662 is a preferable and potential biomarker for diagnosis of CD.

### 3.4 Detailed annotation for interaction between circRNA and miRNA

**Figure 4:**
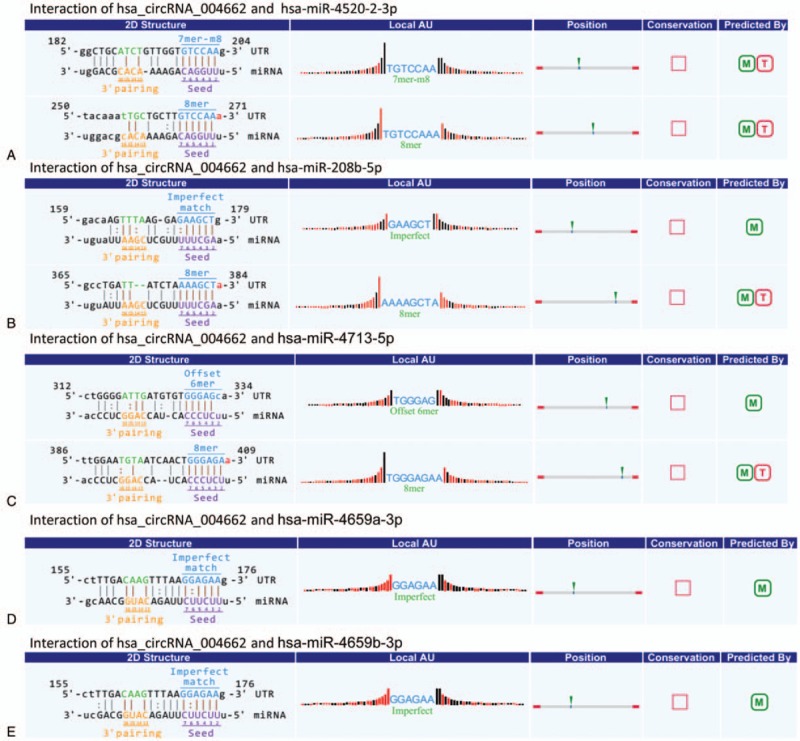
CircRNA_004662 and the 5 most potential target miRNAs predicted. miRNAs that may bind to circRNA were predicted by TargetScan and miRanda. circRNA = circular RNA, miRNA = microRNA.

To reveal which biological process the discovered circRNAs may participate in, miRNAs that may bind to circRNA were predicted by TargetScan and miRanda. The 4 significantly differentially up-regulated circRNAs in CD patients verified were annotated based on the interaction between circRNA and miRNA. The results have been listed in Table 4. The circ_004662 is predicted to harbor hsa-miR-4520-2-3p, hsa-miR-208b-5p, hsa-miR-4713-5p, hsa-miR-4659a-3p, hsa-miR-4659b-3p, with different seed region types (i.e., 8mer,7mer-m8, offset 6mer, and imperfect respectively) (showed in Fig. 4).

### 3.5 CircRNA_004662–microRNA-mRNA network

**Figure 5:**
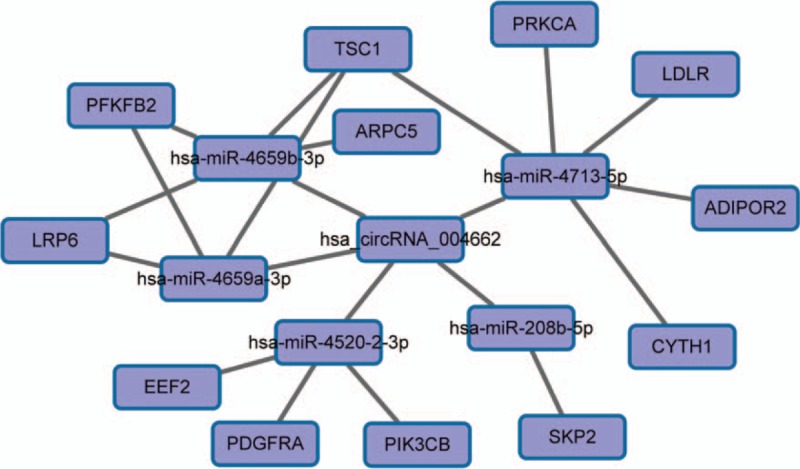
Network of circRNA_004662-miRNA-mRNA. circRNA = circular RNA, miRNA = microRNA.

Target mRNAs of 5 predicted miRNAs which may bind to circRNA_004662 were analyzed by miRDB_V5.0 and TaregetScan7.1. A circRNA_004662–miRNA-mRNA network was built by cytoscape. The results have been shown in Fig. 5. The predicted 5 miRNAs appeared to be correlated with mammalian target of rapamycin (mTOR) pathway.

## 4 Discussion

It is well-known that the stability of circRNAs is higher compared with linear RNAs. The special circular configuration of circRNAs formed by 5′ to 3′-phosphodiester bond leads to its resistance to exonuclease.^[10]^ CircRNAs may be served as potential biomarkers in multiple diseases because of their unique properties.

Functional studies on circRNAs were facilitated by the well-established roles of miRNAs and the next-generation sequencing. CircRNAs can act as microRNA sponges competing miRNAs, regulators of transcription and splicing, adaptor for protein–protein interaction, and participating in ribosomal RNA processing.^[25,26]^ Several studies suggested that dysregulated expression of circRNAs played roles in the development of various types of cancer and particular autoimmune diseases. Recent studies indicated that circRNAs can be served as predictive or diagnostic biomarkers for CRC,^[11]^ breast cancer, RA, pre-eclampsia, and hepatic carcinoma.^[11–15,17,19]^ However, few studies have focused on the roles that circRNAs may play in IBD development.

In our research, a microarray analysis was performed to study the expression profiles of circRNAs in PBMCs of CD patients and HCs. Here, 155 up-regulated circRNAs and 229 down-regulated ones with statistical significance were detected. The differential expression of 5 up-regulated circRNAs were validated by RT-PCR and qPCR in PBMCs. We identified that circRNA_004662 may be a preferable diagnostic biomarker of CD and might be a novel candidate for differentiating CD from UC. CircRNA_004662 is an exonic circRNA that can be spliced from superoxide dismutase 2 (SOD2). SOD2 transforms toxic superoxide to clear mitochondrial reactive oxygen species (mROS), protecting against cell death.^[27]^ Thus, *SOD2* plays important anti-apoptotic roles against ionizing radiation, oxidative stress, and inflammatory cytokines.^[28]^ Expression of *SOD*2 gene is essential for mROS detoxification. In addition, the multidrug resistance-1 (*MDR*1) gene is negatively correlated with expression of *SOD*2. It has been recently reported that *MDR*1 deficiency, which impaired mitochondrial homeostasis, could increase intestinal inflammation response.^[29,30]^ Therefore, we can expect that circRNA_004662 may participate in the functional regulation of the *SOD*2 or *MDR*1.

Given the roles of circRNAs as miRNA “sponges” and gene regulators,^[31]^ we investigated the top 5 predicted miRNA targets paring to circRNAs, and identified that circ_004662 may target hsa-miR-4520-2-3p, hsa-miR-208b-5p, hsa-miR-4713-5p, hsa-miR-4659a-3p, and hsa-miR-4659b-3p, and thus competitively sequester miRNA activity. The predicted 5 miRNAs appeared to be correlated with mTOR pathway.

Previous research has identified that miR-4520a maybe participate in autophagy by regulating Ras homolog enriched in brain (RHEB)/mTOR signaling.^[32]^ Meanwhile, mTOR signaling pathway is involved in the activation and proliferation of T cells. In monocytes/macrophages, mTOR restricts proinflammatory and promotes anti-inflammatory responses.^[33]^ MiR-208 may activate Wnt/β-catenin signaling pathway through targeting Nemo-like kinase (NLK), a Wnt/β-catenin signaling inhibitor.^[34]^ In IBD, Wnt/β-catenin signaling has a multifunctional role in epithelial stem cell identity, proliferation, and epithelial homeostasis regulation.^[35]^ Thus, we conceive that circ_004662 may act as miRNA sponges and be involved in the pathogenesis of CD.

Certain limitation in our study should not be neglected. First, the number of subjects is not large enough, which might correspondingly lead some data deviation. Meanwhile, a more diverse species of disease control group (e.g., intestinal Behcet disease, intestinal tuberculosis [ITB], and other non-IBD enteritis) is also needed to make the conclusion more applicable. Second, we identified the miRNA-circRNA interrelations only by functional analysis, not by verified experiments. Third, because circRNAs are characterized by circular junctions, and the PCR amplicon for the detection of circRNAs using divergent primers spans the backsplice junction of circRNAs, which may make unbiased detection of circRNAs possible. However, without strategies to ensure specific features peculiar to circRNAs, a certain extent of false positives are unavoidable.^[36,37]^ Herein, further researches are still needed in future.

In this study, for the first time, we attempted to detect the differences on circRNAs’ expression level in PBMCs between CD patients and HCs. Results of both microarray analysis and validation performed by RT-PCR and qPCR suggest that circRNAs in PBMC are valuable for diagnosis of CD. In addition, circRNA_004662 may be a preferable diagnostic biomarker of CD. Besides, circRNA_004662 also might be a novel candidate for differentiating CD from UC. Nevertheless, further research is still desired to explore the molecular mechanisms of circRNA_004662 in the development of CD.

## Author contributions

All authors read and approved this final manuscript.

**Conceptualization:** Juan Yin, Zhi Pang.

**Data curation:** Tong Hu.

**Funding acquisition:** Meifen Li, Zhi Pang.

**Investigation:** Yulan Ye, Zhi Pang, Tong Hu.

**Methodology:** Juan Yin, Ping Li.

**Project administration:** Juan Yin

**Resources:** Tong Hu.

**Software:** Juan Yin, Ping Li.

**Supervision:** Lijuan Xu.

**Validation:** Lijuan Xu, Ping Li.

**Visualization:** Meifen Li

**Writing – original draft:** Juan Yin.

**Writing – review & editing:** Yulan Ye and Zhi Pang.
